# Mesenteric Fibrosis in Neuroendocrine Neoplasms: a Systematic Review of New Thoughts on Causation and Potential Treatments

**DOI:** 10.1007/s11912-025-01668-0

**Published:** 2025-04-11

**Authors:** Ariadni Spyroglou, Odysseas Violetis, Konstantinos Iliakopoulos, Antonios Vezakis, Krystallenia Alexandraki

**Affiliations:** 2nd Department of Surgery, Aretaieio Hospital, National and Kapodistrian University of Athens, Vas. Sofias 76, 11528 Athens, Greece

**Keywords:** Mesenteric Fibrosis, Neuroendocrine Neoplasms, Small Intestine, Carcinoid Syndrome

## Abstract

**Purpose of Review:**

Mesenteric fibrosis (MF) is a hallmark of small intestinal neuroendocrine neoplasms (SI-NEN) and is frequently associated with significant morbidity due to related complications such as intestinal obstruction, ischemia, and cachexia.

**Recent Findings:**

Herein we performed a systematic review to discuss the development of MF in SI-NEN. The pathophysiological mechanisms acknowledged as causative for the development of MF include the major components of the tumor microenvironment, such as fibroblasts, endothelial and immune cells and the extracellular matrix, which are involved in a complex interplay activating several signaling pathways that promote profibrotic factors and induce both a desmoplastic reaction and tumor proliferation. Surgery remains the mainstay of treatment, while several medical management options of MF complicating SI-NEN available present rather limited efficacy.

**Summary:**

MF is a frequent characteristic of SI-NEN that requires particular attention and targeted management to avoid complications.

## Introduction

Small-intestinal neuroendocrine neoplasms (SI-NEN) are rare epithelial tumors originating from the neuroendocrine cells of the small bowel. Interestingly, their incidence increased over the past decades, and, as these are indolent neoplasms, they can frequently present at advanced disease stages [1]. SI-NEN originate from enterochromaffin cells of the jejunum and terminal ileum and their symptoms arise either due to their local development, i.e. abdominal pain and diarrhea, or due to the hormonal production, resulting in the carcinoid syndrome (CS). CS is defined by chronic diarrhea and/or flushing together with documented elevated serum serotonin or its metabolite 5-hydroxyindoleacetic acid (5HIAA) [2].

Carcinoid syndrome refers to the symptoms caused by the release of a large variety of humoral factors most commonly biogenic amines, such as serotonin, histamine, tachykinins, kallikrein, prostaglandins, growth factors and other polypeptides secreted by these tumors [3]. CS is a characteristic feature of SI-NEN, and particularly in the case of liver metastases of these midgut tumors, since the biogenic amines secreted from the neoplasm escape the inactivation in the liver and circulate out of the portal vein system [4]. Some of these factors are also responsible for the appearance of a particular form of desmoplastic reaction, the mesenteric fibrosis (MF). The association between CS and fibrosis was first described in 1958, in a case report of scleroderma in association with functioning CS [5]. Although skin fibrosis remains a rare manifestation of CS, MF of the surrounding tissue of is more frequently present in the case of mesenteric lymph node metastases. Mesenteric lymph node metastases are frequently present at diagnosis and can grow inducing MF, further entrapping the superior mesenteric vessels. MF occurs in up to 50% of SI-NEN and can lead to intestinal obstruction, mesenteric ischemia or abdominal pain and malabsorption [6, 7]. Interestingly the presence of MF does not usually co-exist with the occurrence of carcinoid heart disease (CHD) and, thus, patients with MF rarely suffer simultaneously from fibrosis of the right heart valves [8].

To date, neither the complex pathogenetic mechanisms of MF, nor the appropriate and effective treatment options are fully elucidated. Herein we perform a narrative review of the literature on the pathogenetic mechanisms, clinical and diagnostic challenges and the treatment options so far available on MF in SI-NENs.

## Methodology

**Fig. 1:**
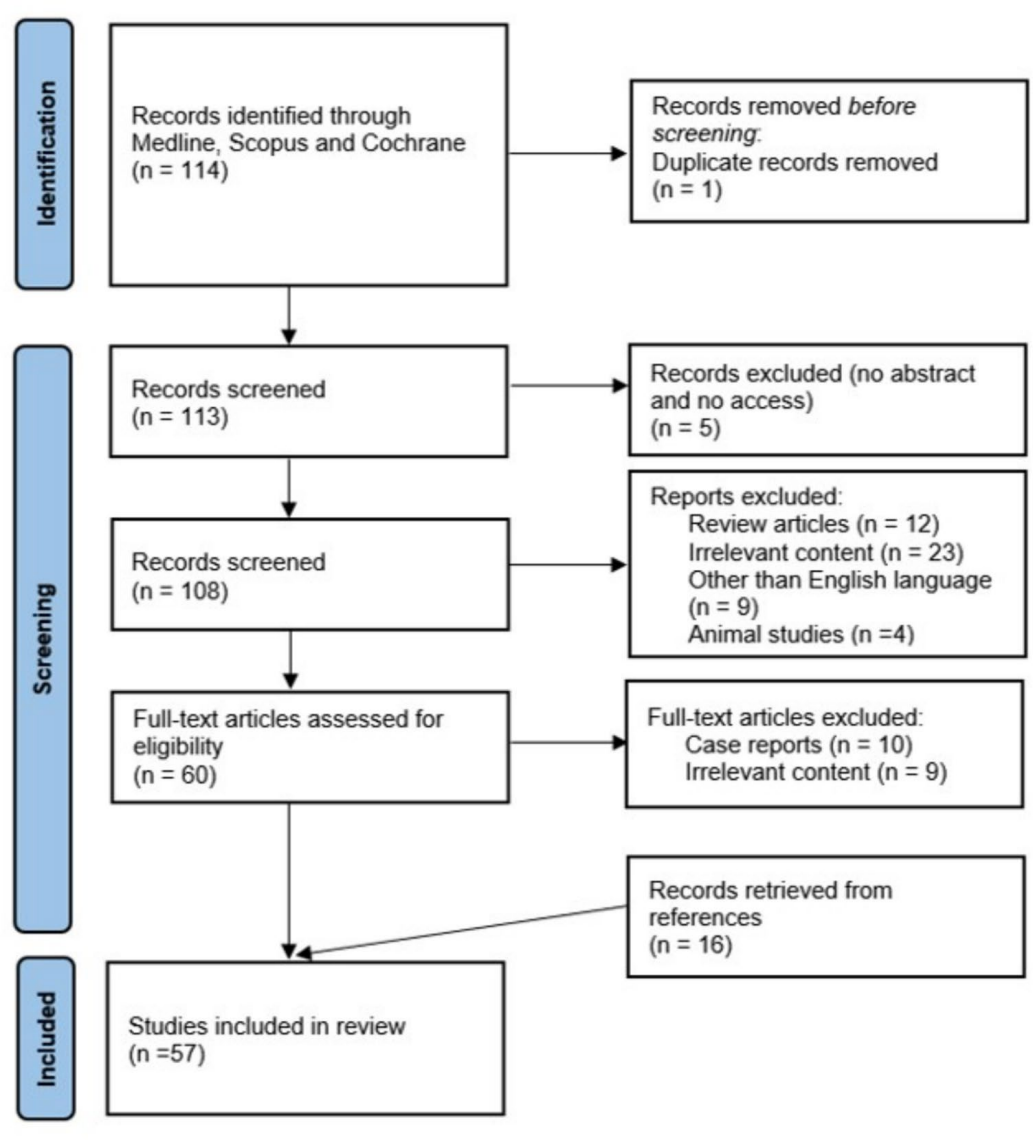
PRISMA flow diagram summarizing the screening and selection process of the studies

We reviewed publications available in PubMed, Cochrane Library and Scopus from the initiation of the databases until the 24th November 2024 and could retrieve 113 articles for review. The PRISMA flow diagram can be found in Fig. 1. The search strategy of this systemic review included following terms: ("neuroendocrine tumour"[All Fields] OR "neuroendocrine tumors"[MeSH Terms] OR ("neuroendocrine"[All Fields] AND "tumors"[All Fields]) OR "neuroendocrine tumors"[All Fields] OR ("neuroendocrine"[All Fields] AND "tumor"[All Fields]) OR "neuroendocrine tumor"[All Fields] OR ("carcinoma, neuroendocrine"[MeSH Terms] OR ("carcinoma"[All Fields] AND "neuroendocrine"[All Fields]) OR "neuroendocrine carcinoma"[All Fields] OR ("neuroendocrine"[All Fields] AND "carcinoma"[All Fields])) OR ("malignant carcinoid syndrome"[MeSH Terms] OR ("malignant"[All Fields] AND "carcinoid"[All Fields] AND "syndrome"[All Fields]) OR "malignant carcinoid syndrome"[All Fields] OR ("carcinoid"[All Fields] AND "syndrome"[All Fields]) OR "carcinoid syndrome"[All Fields])) AND ("retroperitoneal fibrosis"[MeSH Terms] OR ("retroperitoneal"[All Fields] AND "fibrosis"[All Fields]) OR "retroperitoneal fibrosis"[All Fields] OR (("mesenterical"[All Fields] OR "mesenteritis"[All Fields] OR "mesentery"[MeSH Terms] OR "mesentery"[All Fields] OR "mesenteric"[All Fields]) AND ("fibrosi"[All Fields] OR "fibrosing"[All Fields] OR "fibrosis"[MeSH Terms] OR "fibrosis"[All Fields] OR "fibrose"[All Fields] OR "fibroses"[All Fields])) OR ("peritoneal fibrosis"[MeSH Terms] OR ("peritoneal"[All Fields] AND "fibrosis"[All Fields]) OR "peritoneal fibrosis"[All Fields])). Articles were independently evaluated by two of the authors (AS and KIA) for their relevance to the subject of the review. This review was performed according to the PRISMA guidelines for reporting (PROSPERO ID 1001214).

## Results

### Pathophysiology of Mesenteric Fibrosis in Small-intestinal Neuroendocrine Neoplasms

#### Tumor Microenvironment

The potential of tumor invasion and metastasis are mainly attributed to the tumor microenvironment (TME). TME consists of stromal, endothelial, inflammatory cells and extracellular matrix (ECM), in the case of SI-NENs, it is also involved in the development of fibrosis. Desmoplastic reaction is considered as a negative prognostic factor in malignancy. The interplay between tumor cells and surrounding TME is considered responsible for the TME remodeling, which, in turn, enables tumor progression. The local microenvironment in SI-NEN is particularly characterized by a pronounced desmoplastic reaction with limited leucocyte infiltration. Several paracrine amines, peptides and growth factors are involved in this complex process enabling communication of tumor cells with the surrounding stromal cells and promoting the synthesis of profibrotic factors [1, 9].

One of the main factors considered as the driver for the development of MF in SI-NEN is the bioamine serotonin (or 5-hydroxytryptamine, 5-HT). Systemic serotonin secretion by SI-NEN is quantified by the measurement of its metabolite 5HIAA in the urine. However, as local tissue serotonin levels do not always correlate with urinary 5HIAA levels, a paracrine role of this molecule in the development of MF has been suggested. The serotonin role on induction of cardiac fibrosis was confirmed in an in vivo study by Gustafsson et al., in rats that developed heart valve disease after long-term serotonin administration, but for MF no such in vivo experiments are available [10]. In an in vitro study by Svejda et al., serotonin stimulated the fibroblast-like HEK293 cell line proliferation, and synthesis of transforming growth factor β (TGFβ), connective tissue growth factor (CTGF) and fibroblast growth factor (FGF2), whereas administration of a serotonin receptor (5-HT2B) antagonist decreased serotonin and profibrotic growth factor synthesis and secretion [11]. In a study by Blazevic et al., a decreased expression of serotonin-metabolizing enzymes in the stroma of fibrotic mesenteric metastases has been documented, with lower levels of monoamine oxidase A (MAO-A), a key enzyme of serotonin catabolism, in the tumor cells of SI-NEN patients with MF [12].

#### Fibroblasts

Fibroblasts are one of the major cell types surrounding a tumor mass, and in their activated form, also known as cancer-associated fibroblasts (CAF) play an important role in tumor progression. CAFs are characterized by expression of the α-smooth muscle actin (αSMA) and SI-NENs display in fact a high expression of αSMA, suggesting an important role in SI-NEN growth [13]. CAFs in SI-NEN induce the desmoplastic reaction through autocrine/paracrine action of several growth factors [14]. Chaudhry et al., had documented the expression of TGFβ, FGF2, and platelet derived growth factor (PDGF) in NEN of the digestive tract assuming both a stimulation of the tumor and the stromal cell growth and the respective development of a desmoplastic reaction [14, 15]. In vitro experiments have shown that fibroblasts stimulated from the neuroendocrine cell line BON1 secrete TGFβ, whereas further experiments with the co-culture of the SI-NEN cell line KRJ1 and the fibroblast-like cell line HEK293 promotes the release of several growth factors, such as CTGF, FGF2 and TGFβ [11]. TGFβ secreted by SI-NEN induces CTGF expression from both tumoral cells and stromal fibroblasts and both growth factors stimulate collagen synthesis in activated myofibroblasts (now CAF, also known as stellate cells), expressing αSMA [16]. Interleukin-6 (IL-6) and chemo-attractant protein 1 (MCP-1) are secreted by CAF, inducing tumor proliferation [17]. CTGF promotes fibroblast proliferation, migration, adhesion and ECM formation and is highly expressed in tumoral cells adjacent to the stroma [13, 18]. Bone morphogenetic protein 4 (BMP4), a member of the TGFβ superfamily was further found highly expressed in SI-NEN with pronounced fibrosis, rendering this molecule also suspect for the pathogenesis of MF [19]. Finally, fibroblasts nerve growth factors (NGF), regulating angiogenesis [19].

#### Endothelial Cells

NENs are highly vascularized tumors, reminiscent of the physiological vascular supply of endocrine tissues, and present the paradox, that well-differentiated neoplasms display higher vascular density than poorly differentiated tumors [20]. The vascular endothelial growth factor (VEGF) system, consisting of VEGF and its receptors VEGFR1 and VEGFR2, is highly expressed in these endocrine neoplasms exerting a relevant paracrine action promoting neovascularization. An aberrant activation of the mammalian target of rapamycin (mTOR) signaling pathway is also acknowledged as a factor promoting angiogenesis in these neoplasms [17]. Interestingly, VEGF inhibition has a low effect on limiting tumor growth implying a resistance of these neoplasms. Besides an intrinsic resistance of these neoplasms, as observed by treatment with the VEGF inhibitor sunitinib, an acquired resistance to antiangiogenic treatment can also appear after an initial effective inhibition of angiogenesis. In this case, the resulting hypoxia stimulates the release of hypoxia-inducible factor 1α (HIF1A), which, in turn, induces alternative proangiogenic factors [18]. Further proangiogenic factors include FGF2 and PDGF. FGF2 stimulates endothelial cell proliferation, but its role on MF remains uncertain, as FGF2 expression was comparable in patients with SI-NEN in comparison to controls but also independent from the extent of MF [19]. PDGF, secreted from endothelial cells, also promotes vascular formation and accentuates NEN proliferation. Interestingly, and similar to the CTGF expression, also PDGF expression is higher in tumor cells adjacent to the stroma [21]. Furthermore, PDGF receptor β expression was found pronounced in stromal cells of metastases [14, 22].

#### Immune Cells

Immune cells are another major component of the TME, with B- and T-lymphocytes, natural killer cells (NK), macrophages, infiltrating the tumor. Interestingly, in SI-NEN the leucocyte infiltration is significantly lower than in other malignancies [17], with T-regulatory lymphocytes, infiltrating in particular metastases, possibly inhibiting the anti-tumor effect of other T-lymphocytes [23]. NK cells are known to inhibit TGFβ profibrotic activity. Substance P (SP), a tachykinin secreted by SI-NEN antagonizes the anti-fibrotic action of NK cells, possibly contributing to MF [24]. Furthermore, NEN tumors display a disturbed NK cell function with deficient interferon (IFN)-α response [17]. Tumor-associated macrophages (TAM) support tumor development, by suppression of the adaptive immune system and stimulate the secretion of profibrotic factors such as TGFβ, promoting fibrosis. In SI-NEN TAMs strongly express TGFβ and PDGF [22]. The expression of the immune checkpoint molecule programmed death-ligand 1 (PDL1) in SI-NEN is rather low. In line with this observation, with the application of two different monoclonal antibodies, we have recently shown no positive PDL1 expression in SI-NEN [25]. However, PD1 antibodies are currently under investigation for their effectiveness in NEN progression [26]. Unlike this, in a study by Rodrigez et al. a significant IgG4 expression in plasma cells of mesenteric tumor deposits in SI-NEN patients was found suggesting a possible interrelation of MF with the IgG4-related disease [27].

#### Extracellular Matrix (ECM)

**Fig. 2:**
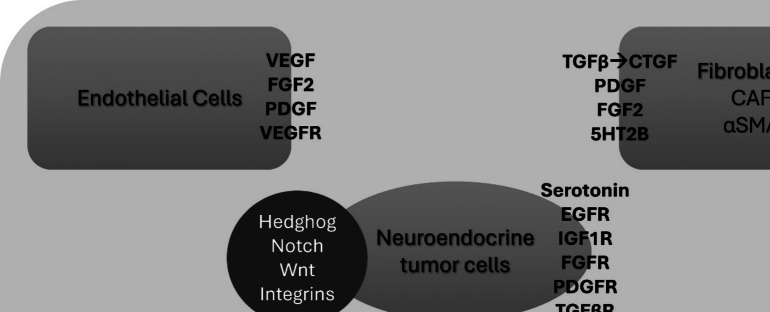
Components affecting the tumor microenvironment and contributing to mesenteric fibrosis in SI-NEN. 5HT2B: serotonin receptor 2B, αSMA: α-smooth muscle actin, CAF: cancer-associated fibroblasts, CTGF: connective tissue growth factor, EGF: epidermal growth factor, EGFR: epidermal growth factor receptor, FGF2: fibroblast growth factor, FGFR: fibroblast growth factor receptor, IFNα: interferon-α, IGF1: insulin-like growth factor 1, IGF1R: insulin-like growth factor 1 receptor, LC: lymphocytes, MMPs: matrix metalloproteinases, NK: natural killer cells, PDGF: platelet-derived growth factor, PDGFR: platelet-derived growth factor receptor, SP: substance P, TAM: tumor associated macrophages, TGFβ: transforming growth factor β, TGFβR: transforming growth factor β receptor, VEGF: vascular endothelial growth factor, VEGFR: vascular endothelial growth factor receptor

ECM consists of polypeptides and polysaccharides necessary for structural integrity and tissue homeostasis. ECM remodeling plays an important role in tumor development, invasion and finally metastasis. Heparan and chondroitin proteoglycans bind several growth factors and chemokines and their differential regulation during disease progression in SI-NEN might affect the development of fibrosis [28]. Matrix metalloproteinases (MMP) are normally involved in the degradation and remodeling of matrix and promote angiogenesis, and their absence correlated with an aggressive phenotype in an in vivo model of pancreatic NEN (PanNEN), but no data are available on their role in SI-NEN [18]. Collagen III, a further component of ECM, accumulates in proximity to CAFs, as product of these fibroblasts, also contributing to fibrosis [16]. Components of the extracellular matrix further include profibrotic growth factors, such as serotonin, CTGF, TGFβ, PDGF, FGF2, with their role in MF pathogenesis already discussed. Further growth factors, such as insulin like growth factor (IGF-1), epidermal growth factor (EGF) and TGFα might also present an autocrine action relevant for tumor proliferation, as their receptors are present in NEN. Still, their role in MF remains unclear [18]. Recently, sexual dimorphism was observed in the development of MF, with premenopausal women presenting reduced MF and better prognosis, and this fact correlating with an increased estrogen receptor (ER) α and AR expression in the SI-NEN microenvironment [29] (Fig. 2).

#### Signaling Pathways

Well-known pathways (Hedgehog, Notch, Wnt/β-catenin, integrins), implicated both in fibrotic diseases and cancer have also been studied in SI-NEN. In an immunohistochemical study (IHC) of SI-NEN, the expression of Sonic Hedgehog ligand was found positive in almost three quarters of the patients [30]. In a further study focusing on the Notch pathway, no significant difference in the transcription of *NOTCH1, NOTCH2, NOTCH3, HES1, HEY1* and *HEY2* was documented in a series of 8 SI-NEN, but upregulated *ASCL1* transcription was found in half of the tumors [31]. However, IHC staining of NOTCH1 and HES1 on a tissue micro-array of 31 SI-NEN was negative [32]. In the case of the wnt/β-catenin signaling pathway, no correlation of the severity of MF with the β-catenin expression was found [19], whereas a further study showed only normal membranous expression of β-catenin in SI-NEN [30]. In an in vitro co-culture model of SI-NEN cells KRJ-I with HEK293 conditioned media the integrin subunit alpha v (ITGAV) was significantly upregulated, whereas in HEK293 cells with KRJ-1 conditioned media Metalloproteinase 8 (MMP8), TGFβ3 and the integrin ITGB8, all with a role on fibrosis development, were significantly downregulated. The activation of the integrin pathway was further validated in a cohort of SI-NEN, where a significant upregulation of the integrin pathway genes TGFβ1, collagens COL1A1, COL3A1, fibronectin 1, and integrins ITGAV, and ITGAX was documented [33].

#### Fibrosome and Other Profibrotic Molecules

Laskaratos et al., recently introduced the definition of fibrosome, including a subset of circulating transcripts from the gene molecular signature of the NETest with known roles in fibrosis, namely: CTGF, the complement regulator CD59, amyloid precursor-like protein 2 (APLP2), frizzled homologue 7 (FZD7) and BNIP3L and documented that the presence of APLP2, BNIP3L, CD59 and CTGF transcripts could independently predict the presence of MF in SI-NEN at a statistically significant level [34]. However, in a further IHC study, no correlation of APLP2, BNIP3L, or CD59 tissue expression with the presence of MF could be demonstrated [35]. In a proteomic analysis comparing samples from SI-NEN patients with and without MF, differential protein expression was only documented in the mesenteric stroma, where Collagen α1(XII) (COL12A1) and complement C9 expression had higher abundance in MF samples [36]. Finally, in a biomarker panel study by Graf et al., differential gene expression between SI-NEN patients with or without MF included increased COMP and COL11A1 gene expression in the stroma of MF patients, and decreased HMGA2, COL6A6, and SLC22A3 expression in lymph node metastases of MF patients, with COMP being strongly associated with the known profibrotic factor TGFβ [37].

### Clinical Presentation and Diagnosis of Mesenteric Fibrosis in Small-intestinal Neuroendocrine Neoplasms

Due to their indolent nature, SI-NEN are often diagnosed at advanced stages, and their clinical manifestations frequently occur due to the extensive MF adjacent to local lymph node metastases and locoregional mesenteric tumor deposits. Extensive MF in SI-NEN patients can cause acute symptoms due to mesenteric vessel obstruction leading to intestinal ischemia, and intestinal obstruction. Abdominal pain, worsening of diarrhea and ascites are the main acute symptoms of MF. Postprandial abdominal pain and diarrhea can further induce malabsorption and lead to nutritional deficiencies, in particular of fat-soluble vitamins, dehydration and electrolyte imbalances [9]. Furthermore, MF can extend to the retroperitoneum. Here, it is hypothesized that biogenic amines escaping liver inactivation are the main triggers for the development of retroperitoneal fibrosis, leading to obstructive uropathy, hydronephrosis up to renal failure at later disease stages [38].

The radiological findings in the case of MF in SI-NEN are rather peculiar. Pantongrag et al., described a classic trias of signs characteristic of SI-NEN including a calcified mesenteric mass, radiating strands, and adjacent bowel-wall thickening. A mesenteric mass with radiating strands of soft-tissue on CT imaging is now considered a pathognomonic feature of SI-NEN related MF [39]. Further radiological manifestations suggestive of MF in a cohort of SI-NEN patients include mesenteric vessel encasement, presence of hepatic metastases and large hepatic tumor burden, mesenteric mass with coarse or fine calcification, “indrawing” of tissues and “misty” mesentery [40, 41]. The presence and extent of lymph node metastases and of MF in SI-NEN patients play an important role for surgical planning, so that radiological assessment should be optimized. Therefore, functional imaging with ^68^Ga-DOTATOC-PET/CT can improve the detection of lymph node metastases in MF. While ^18^F-FDG-PET/CT is only helpful in the visualization of aggressive NEN, in a case report, 18F-FDG uptake was documented in a retroperitoneal fibrotic mass of a patient with a carcinoid tumor [42].

The histological assessment of MF is based on the section of the surgical specimen with the highest amount of fibrous tissue. Histologically, SI-NEN fibrosis presents hypocellular, with prominent hyalinization and development of foci of calcification. Histological distinction between positive lymph nodes and MTD can be difficult in many cases and irregular shape and nerve/vessel entrapment can serve as a marker of MTD [43].

### Surgical Management of Mesenteric Fibrosis

The fibrotic process, driven by tumor-associated desmoplastic reactions, leads to mesenteric retraction and vascular compromise, often necessitating surgical intervention. Surgery is often required to manage symptoms and prevent complications arising from MF. Palliative surgery is indicated for patients experiencing intestinal obstruction, ischemic complications, and severe abdominal pain [44]. Similarly, it has been shown that 82% of patients undergoing laparotomy for midgut carcinoid tumors experience symptom relief, supporting a proactive surgical approach [45]. Despite this, no significant survival benefit has been observed with surgical resection compared to non-surgical management, emphasizing its primarily palliative role. Surgery should aim at a systematic lymphadenectomy avoiding a short bowel syndrome.

#### Surgical Techniques

All localized SI-NENs should undergo surgery with proper lymph node dissection. The definition of surgical resectability should take into account the degree of arterial involvement in lymph-node metastases. According to the classification of Ohrvall and colleagues Stage I consists of tumors located close to the intestine, stage II includes tumors involving arterial branches close to their origin in the mesenteric artery, stage III comprises tumors extending along, without encircling, the superior mesenteric artery trunk, and finally stage IV refers to tumors that extend retroperitoneally, behind or above the pancreas, or grow around the mesenteric artery and involve the origin of proximal jejunal arteries on the left side of the superior mesenteric artery [46].

A combination of primary tumor and mesenteric mass resection is recommended for curative intent and to prevent debilitating MF [47]. The preferred surgical approach aims at a balance between oncologic radicality and preservation of bowel function. A mesenteric-sparing technique is often favored to avoid excessive bowel resection.

Vessel-sparing lymphadenectomy (VS-LA) seems to be preferred over conventional lymphadenectomy (Con-LA) due to its superior postoperative outcomes. VS-LA has shown to result in shorter bowel resections (median 40 cm vs. 65 cm, *p* = 0.007), lower rates of postoperative complications (4% vs. 28%, *p* = 0.02), and comparable oncological outcomes (R0 resection rate: 72% vs. 84%). For this reason, the surgical approach should not be a “pizza pie” approach (including a large intestinal resection), but a retrograde VS-LA [48]. In a recent retrospective study, it has been shown that the resected bowel specimen is shortened by about half, when using this technique [49]. In cases where complete resection is unfeasible due to extensive vascular involvement, intestinal bypass procedures may be performed to alleviate obstruction. However, this approach is reserved for patients with severe symptoms, as it does not provide a survival advantage.

#### Staging and Surgical Feasibility

**Fig. 3:**
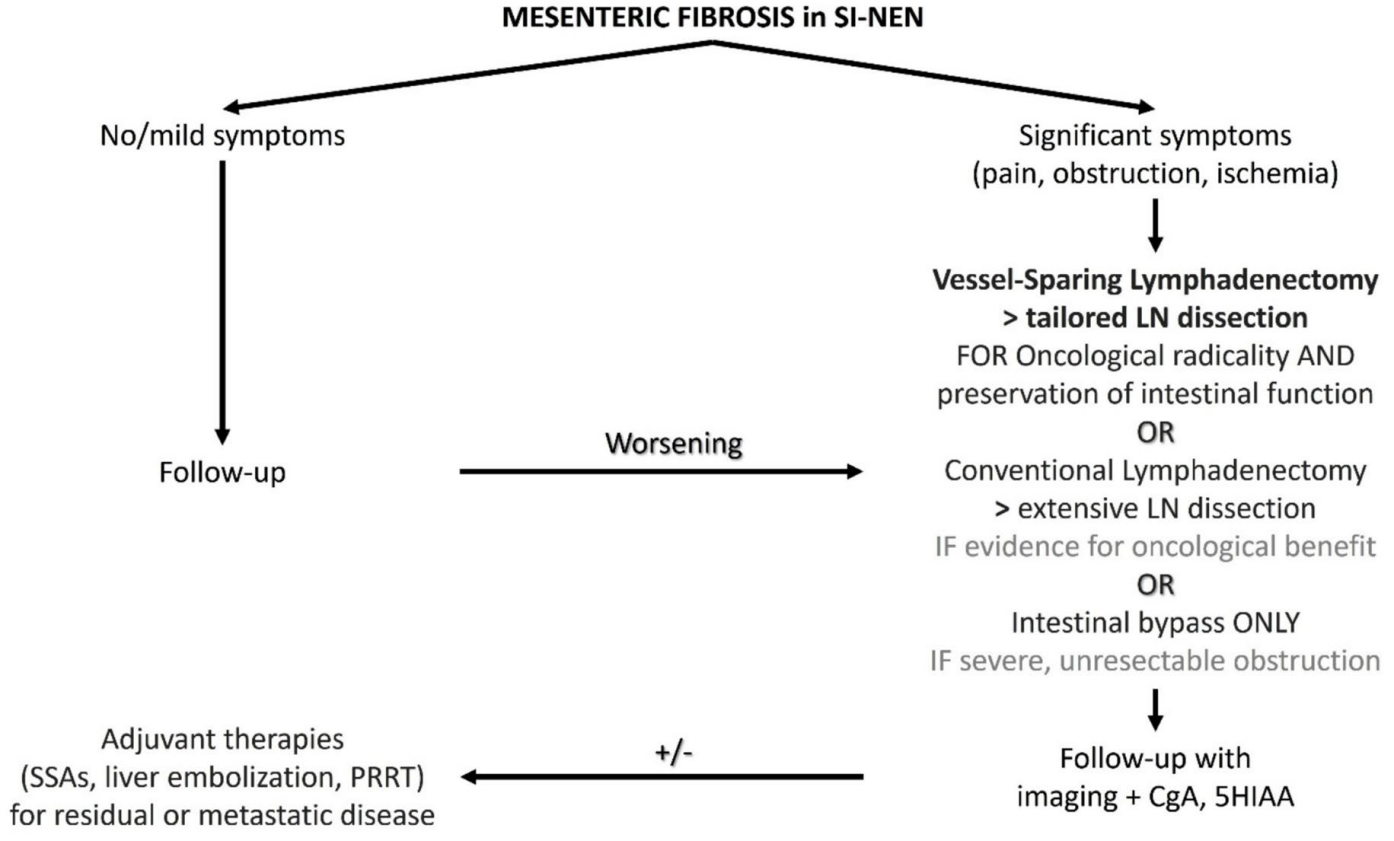
Suggested algorithm for surgical intervention in mesenteric fibrosis in SI-NEN. 5HIAA: 5-hydroxyindoleacetic acid, CgA: Chromogranin A, LN: lymph nodes, PRRT: peptide receptor radionuclide therapy, SSA: somatostatin analogues

In the context of MF resectability, mesenteric disease can be categorized into three subtypes: type A including the resectable mesenteric disease not involving the mesenteric root; type B including a borderline resectable disease with fibrosis near but not encasing major mesenteric vessels; type C including locally advanced, unresectable disease with complete encasement of the superior mesenteric artery (SMA) and vein (SMV). For Types A and B, surgery is generally feasible with experienced surgical teams. Type C lesions, however, may necessitate alternative palliative strategies, such as somatostatin analogs, nutritional support, and palliative bypass [50]. A suggested algorithm for surgical intervention can be found in Fig. 3.

#### Surgical Outcomes

Several studies have assessed the effectiveness of surgical intervention for MF. It is shown that 41.4% of patients exhibited MF, with older age, high 5-HIAA levels, and large mesenteric masses being independent predictors. The authors reported no significant survival benefit from metastasectomy of mesenteric masses or prophylactic surgery, suggesting that surgical intervention should be symptom-driven rather than routinely performed [44]. In contrast, it is demonstrated that mesenteric tumor dissection with vessel preservation significantly improved symptoms and quality of life, even in cases initially deemed inoperable [49]. Similarly, lymphadenectomy should be carefully tailored to balance tumor clearance with bowel preservation, as aggressive resection can lead to complications such as short bowel syndrome [47].

#### Long-term Outcomes and Survival

Median survival after laparotomy was 9 years, with a subset of patients achieving 12-year survival. Notably, survival was independent of surgical intervention in patients with extensive metastases, emphasizing the palliative nature of surgery [45]. In the context of quality of life and functional outcomes, patients who underwent VS-LA had significantly lower postoperative complication rates (4% vs. 28%) and lower incidence of postoperative diarrhea (4% vs. 40%) [48]. This highlights the importance of preserving intestinal length and vascular integrity during surgery. SI-NEN should be operated in high-volume centers of excellence since it was shown that the 90-day mortality after surgery is higher in low-volume centers compared to high-volume hospitals (4% vs. 1%) [51].

### Medical Management of Mesenteric Fibrosis

#### Somatostatin analogues

Somatostatin analogues (SSAs) are considered a first-line therapy for functioning NEN [52, 53]. Nearly 80% of carcinoid tumors express somatostatin receptors (SSTRs). SSAs can attenuate the symptoms of carcinoid syndrome including diarrhea and flushing via their anti-secretory effects [54]. The PROMID and CLARINET studies have proved that first-generation SSAs, the long-acting octreotide and lanreotide exert a stabilizing effect on tumor growth in patients with midgut and gastroenteropancreatic NENs respectively. Pasireotide, a novel SSA showing affinity for all SSTRs, might also be effective in patients with NENs [6]. Treatment with first-generation SSAs reduced urine 5-HIAA levels in approximately 50% of the patients, though, few achieved complete biochemical control whereas only 29% of patients treated with pasireotide showed lower levels. Theoretically, if fibrosis is stimulated, at least, partly by serotonin, SSAs might reduce its formation. Higher 24h urine 5-HIAA levels have been associated with MF, therefore the administration of SSAs could preclude the desmoplastic reaction [55]. Additionally, patients treated with SSAs showed reduced levels of profibrotic growth factors CTGF and TGF levels, which have a central role in fibrosis formation [56]. Fibrosis in other pathological situations including liver, pulmonary and peritoneal sclerosis was seen to be attenuated by the treatment with SSAs [1]. However, since MF is presented in more advanced stages, most patients have already been treated with SSAs, thereby complicating the evaluation of SSAs in fibrosis development [44]. Prospective studies remain to evaluate the effect of SSAs on the treatment and, even, prevention of MF.

#### Serotonin Synthesis Inhibitors and 5-HT Receptor Antagonists

Since serotonin is deemed as the main driver of desmoplastic reaction in SI-NEN, the development of drugs inhibiting its production or its signaling seemed to be more than rational. Serotonin is derived from the amino acid tryptophan. Two enzymic steps are required with the first being, tryptophan hydroxylation by tryptophan hydroxylase (THP) to 5-hydroxytryptophan (5-HTP) and the second, 5-HTP decarboxylation to form serotonin [57]. The peripheral 5-HTP decarboxylation of serotonin was the first target to reduce its synthesis by agents such as phenylacetic acid and α-methyl-dopa. However, they were not successful, as they presented a moderate effect on lowering serotonin synthesis along with serious side effects. The next target was THP, a rate-limiting enzyme in the serotonin synthesis pathway which occurs in 2 isoforms in the human body, THP1 in the enterochromaffin cells causing gastrointestinal (GI) symptoms in CS and THP2 in the central nervous system [58]. Engelman et al. tested this hypothesis using parachlorophenylalanine (PCPA) succeeding in urinary 5-HIAA levels decrease and symptoms improvement. However, universal inhibition of THP precluded psychiatric disorders as adverse effects, including depression, limiting PCPA clinical use [59]. A recently developed oral small-molecule TPH inhibitor (telotristat ethyl) which acts only on the TPH in the periphery without crossing the blood–brain, thus inevitably inhibiting selectively THP1, was assessed in 2 randomized phase III trials (TELESTAR and TELECAST) in patients with CS. Within the TELESTAR trial telotristat ethyl treatment resulted in a reduction in bowel movement frequency along with a significant reduction of urinary 5-HIAA levels. The TELECAST study further confirmed the efficacy and safety of telotristat ethyl [60]. However, long-term complications such as CHD or MF were not assessed. Interestingly, telotristat studied in a mouse model of serotonin-secreting metastasized NEN showed a non-significant decrease, but clinically important, in NT-proBNP levels, a quite sensitive indicator of early CHD, and heart valve fibrosis [61]. Interestingly, telotristat is currently tested under a phase III clinical trial evaluating its role, combined with that of SSA, in controlling CHD in patients with metastatic NEN (clinicaltrials.gov/ NCT04810091).

Aside from the inhibition of 5-HTsynthesis, 5-HT receptor antagonists might reduce MF in SI-NEN. Since the profibrotic effects of serotonin fibroblast growth and fibrogenesis are mediated via the 5-HT1A/B and 5-HT2A/B receptors, relevant drugs acting on these receptors should be potential treatments for CS-associated fibrotic complications [62]. Notably, cyproheptadine, a 5-HT2 antagonist, was found to prevent toxic valvulopathy in an animal model in which intraperitoneal injections of pergolide, a serotonin agonist, were administered [63]. Additionally, cyproheptadine was found to decrease the CS symptoms, namely flushing and stool frequency. The non-selective 5-HT2 receptor antagonist ketanserin also resulted in a decrease in CS symptomatology [64]. However, these molecules have not been specifically assessed for their potential antifibrotic effect [62]. Several preclinical studies demonstrated the preventive effect of terguride, a 5-HT2B/2CR antagonist, on 5-HT-induced heart disease development [65]. These results, though preliminary, suggest a possible therapeutic agent which may be worthy of further investigation, particularly because terguride has been safely administered to scleroderma patients leading to a reduction of skin fibrosis [66].

#### Molecular-targeted Therapies

Remarkable recent progress in understanding the pathogenesis of MF has allowed the identification of other mediators, except for serotonin, namely growth factors such as EGF, PDGF, TGFα, TGFβ and CTGF [24]. Therefore, targeting their receptors or their signaling pathways by tyrosine kinase inhibitors (TKIs) might be efficient in reducing SI-NEN progression and the development of fibrotic complications. Multi-targeted TKIs such as sunitinib, surufatinib, pazopanib, sorafenib, axitinib, saracatinib, lenvatinib, and cabozantinib have shown promising responses in patients with advanced PanNEN [26]. However, TKIs demonstrated less efficacy in patients with advanced GI-NEN, even when their impact on MF and CHD has not been specifically addressed. Research focuses on VEGF, PDGF and their receptors, as hypervascularity is considered a hallmark of NEN aiming at their anti-tumor properties [67]. Various TKIs are currently under extensive investigation as potential antifibrotic agents for systemic sclerosis, idiopathic pulmonary fibrosis and other fibrotic disorders [68]. Since the signaling pathways involved in the development of fibrosis present similarities between SI-NEN and other fibrotic diseases, it would be essential in the future to study also the effects of these compounds on tumor microenvironment and growth factors secretion. Bevacizumab, a monoclonal antibody against VEGF-A, is also applicable to the SI-NEN management, either alone or as in combination with SSA, sorafenib or systematic chemotherapy, but its effectiveness on MF has not been clarified so far [69].

Upregulation of phosphatidylinositol 3-kinase (PI3K)-Akt-mTOR pathway, which has a physiological role in protein synthesis, cell growth and cell metabolism, has been identified as a crucial component in NEN development, which can occur either through direct activation or by the downregulation of natural inhibitors, notably phosphatase and tensin homolog (PTEN). mTOR has been the first target of the pathway [70]. The treatment of advanced well-differentiated PanNEN and SI-NEN with everolimus, mTORC1 inhibitor, was correlated with a significantly improved progression-free survival (PFS) in the RADIANT trial program, even though it is not known to affect the desmoplastic reaction in the mesentery [6]. Unfortunately, its response seems not to be durable, due to the non-sustained inhibition of mTORC1 signaling and/or activation of mTORC2. Notably, mTOR inhibitors were shown to decrease the expression of 5-HT receptors on valve cells, hence, they can be used for the prevention of CHD [65]. Everolimus treatment has produced conflicting evidence, as it can function both as profibrotic in some situations and antifibrotic agent in others [71, 72].

#### Peptide Receptor Radionuclide Therapy

Peptide receptor radionuclide therapy (PRRT), a radiolabeled SSA therapy, is an effective treatment for SI-NEN. In a multinational phase III randomized, double-blind, prospective clinical trial (NETTER trial), patients with advanced, progressive, SSTR-positive midgut NEN received either ^177^Lu-DOTOTATE plus low-dose octreotide LAR (30 mg) or high-dose octreotide LAR (60mg). The study showed that ^177^Lu-DOTA-TATE significantly prolonged PFS compared to octreotide-LAR alone with a significant clinical improvement [73]. The impact of PRRT on MF is a relatively open field. Retrospective studies advocate that desmoplastic reaction does not morphologically respond to PRRT treatment. Nevertheless, PRRT may aid in stabilizing mesenteric metastases, thereby preventing mesenteric mass-related symptoms. Recently, a multicenter retrospective study showed clinical improvement in almost half of patients with related symptoms, which persisted (partially or completely) after the end of PRRT, suggesting a possible long-term clinical impact of PRRT [74]. Similarly, a recent case-series study presented two patients with SI-NEN with MF who exhibited significant improvement after ^177^Lu-DOTA-TATE PRRT [75]. On the other hand, there are concerns for an increased risk of fibrotic complications during PRRT treatment, perhaps because of radiation-provoked inflammation, for which a course of corticosteroids prior to treatment is believed to avert GI complications [76]. Of note is also the fact that PRRT can induce a carcinoid crisis through acute release of stored bioamines upon tumor lysis and even lead to acute cardiac failure. In this context, all patients should be screened for CHD prior to PRRT, whereas appropriate management for the prevention of a carcinoid crisis during PRRT treatment includes corticosteroids, antiemetics, SSAs and antihistamine drugs [77]. Further studies are needed to elucidate the clinical value of PRRT for patients with SI-NETs and MF.

#### Other Anti-fibrotic Agents

The renin-angiotensin system (RAS) seems to be involved in tumor growth, by inducing angiogenesis and tumor proliferation [78] as well as in the pathogenesis of fibrosis, mainly due to its ability to activate TGFβ signaling, a key feature of fibrosis. Indeed, it has been reported that angiotensin-converting enzyme inhibitors (ACEis) improve the fibrotic processes in several organs including the heart, lung, liver, and kidney [79]. ARBs and ACEi might improve patient outcome in several cancers, including breast, colorectal, pancreatic, and gastric cancer, but their tumor abilities remain to be elucidated [80]. Notably, in vitro ACE inhibition was shown to reduce BON1 cell proliferation [81]. However, more research is needed to establish the effect of ACEis’ clinical efficacy on patients with SI-NEN and related fibrotic complications. Furthermore, tamoxifen is a synthetic nonsteroidal selective ER modulator (SERM), developed initially for the treatment of breast cancer. It has been used for CS control with varied responses. Tamoxifen is also deemed a treatment option for fibrotic complications of SI-NEN as it inhibits the TGF secretion by fibroblasts. Biasco et al. showed amelioration of fibrosis after a long-term treatment with octreotide and tamoxifen in a patient with a NEN complicated by retroperitoneal fibrosis causing right ureteral obstruction [82, 83]. Recently, in a single-arm phase II trial of tamoxifen in patients with metastatic, progressive NEN with positive expression of ER and/or progesterone receptor (PR), it was shown that it exerts modest efficacy without clinical improvement of presented symptoms in functioning NEN or MF development [83]. IFN is an established treatment for SI-NEN with studies showing its efficacy in tumor progression, symptoms of CS and tumor markers [55]. Apart from its antiproliferative properties, it also demonstrates anti-fibrotic activities. IFN seems to be a potential effective biological agent to treat lung, liver and renal fibrosis [84]. Thus, it could have a role in the treatment of MF, but there is no evidence for that matter. Several side effects are associated with its administration, flu-like symptoms among them, limiting its universal use [85].

## Conclusions

MF is a particular and frequent trait of SI-NEN, mainly complicated by CS, that develops through complex pathophysiological interactions occurring in the TME. MF can cause intestinal obstruction, ischemia, or cachexia and its management can be challenging. Apart from surgical resection, that remains the mainstay of treatment, several medical therapies, in their majority already established in the management of SI-NEN and/or CS, have been tested for their impact on the reduction of MF. Still, their efficacy remains limited and further molecular and clinical studies are needed to guide the development of treatments that could limit the development of MF.

## Key References

- Grozinsky-Glasberg, S.; Davar, J.; Hofland, J.; Dobson, R.; Prasad, V.; Pascher, A.; Denecke, T.; Tesselaar, M.E.T.; Panzuto, F.; Albage, A.; et al. European Neuroendocrine Tumor Society (ENETS) 2022 Guidance Paper for Carcinoid Syndrome and Carcinoid Heart Disease. J Neuroendocrinol 2022, 34, e13146, 10.1111/jne.13146.
  - This study includes the newest ENETS guidelines on carcinoid syndrome.
- Blazevic, A.; Iyer, A.M.; van Velthuysen, M.F.; Hofland, J.; van Koestveld, P.M.; Franssen, G.J.H.; Feelders, R.A.; Zajec, M.; Luider, T.M.; de Herder, W.W.; et al. Aberrant tryptophan metabolism in stromal cells is associated with mesenteric fibrosis in small intestinal neuroendocrine tumors. Endocr Connect 2022, 11, 10.1530/EC-22-0020.
  - This study describes new findings on the generation of mesenteric fibrosis through aberrant tryptophane metabolism.
- Ratnayake, G.M.; Laskaratos, F.M.; Mandair, D.; Caplin, M.E.; Rombouts, K.; Toumpanakis, C. What Causes Desmoplastic Reaction in Small Intestinal Neuroendocrine Neoplasms? Curr Oncol Rep 2022, 24, 1281–1286, 10.1007/s11912-022-01211-5.
  - The study describes the causes of desmoplastic reaction in mesenteric fibrosis.
- Blazevic, A.; Iyer, A.M.; van Velthuysen, M.F.; Hofland, J.; Franssen, G.J.H.; Feelders, R.A.; Zajec, M.; Luider, T.M.; de Herder, W.W.; Hofland, L.J. Proteomic analysis of small intestinal neuroendocrine tumors and mesenteric fibrosis. Endocr Relat Cancer 2023, 30, 10.1530/ERC-22-0237.
  - This is the first study investigating proteomics of small intestinal neuroendocrine tumors and their interrelation to mesenteric fibrosis.
- Gonzalez, R.S.; La Rosa, S.; Ma, C.; Polydorides, A.D.; Shi, C.; Yang, Z.; Cox, B.; Karamchandani, D.M. Debating Deposits, Redux: Substantial Interobserver Agreement Exists in Distinguishing Tumor Deposits From Nodal Metastases in Small Bowel Neuroendocrine Tumors. Arch Pathol Lab Med 2024, 148, 581–587, 10.5858/arpa.2023-0169-OA.
  - This study describes characteristics distinguishing tumor deposits from nodal metastases in small intestine neuroendocrine tumors.
- Bartsch, D.K.; Windel, S.; Kanngiesser, V.; Jesinghaus, M.; Holzer, K.; Rinke, A.; Maurer, E. Vessel-Sparing Lymphadenectomy Should Be Performed in Small Intestine Neuroendocrine Neoplasms. Cancers (Basel) 2022, 14, 10.3390/cancers14153610.
  - This is a comprehensive study explaining the need for vessel-sparing lymphadenectomy in small intestine neuroendocrine neoplasms.
- Lamarca, A.; Bartsch, D.K.; Caplin, M.; Kos-Kudla, B.; Kjaer, A.; Partelli, S.; Rinke, A.; Janson, E.T.; Thirlwell, C.; van Velthuysen, M.F.; et al. European Neuroendocrine Tumor Society (ENETS) 2024 guidance paper for the management of well-differentiated small intestine neuroendocrine tumours. J Neuroendocrinol 2024, 36, e13423, 10.1111/jne.13423.
  - These are the most recent ENETS guidelines on the management of small intestinal neuroendocrine tumors.
- Alexandraki, K.I.; Angelousi, A.; Chatzellis, E.; Chrisoulidou, A.; Kalogeris, N.; Kanakis, G.; Savvidis, C.; Vassiliadi, D.; Spyroglou, A.; Kostopoulos, G.; et al. The Role of Somatostatin Analogues in the Control of Diarrhea and Flushing as Markers of Carcinoid Syndrome: A Systematic Review and Meta-Analysis. J Pers Med 2023, 13, 10.3390/jpm13020304.
  - This study investigates the role of somatostatin analogues in the management of carcinoid syndrome.
- George, J.; Ramage, J.; White, B.; Srirajaskanthan, R. The role of serotonin inhibition within the treatment of carcinoid syndrome. Endocr Oncol 2023, 3, e220077, 10.1530/EO-22-0077.
  - This study investigates the role of serotonin inhibition in the management of carcinoid syndrome.
- Al Mansour, L.; De Mestier, L.; Haissaguerre, M.; Afchain, P.; Hadoux, J.; Lecomte, T.; Morland, D.; Cottereau, A.S.; De Rycke, O.; Tlili, G.; et al. Outcome on Mesenteric Mass Response of Small-Intestinal Neuroendocrine Tumors Treated by (177)Lu-DOTATATE Peptide Receptor Radionuclide Therapy: The MesenLuth Study, a National Study from the French Group of Endocrine Tumors and Endocan-RENATEN Network. J Nucl Med 2024, 65, 258–263, 10.2967/jnumed.123.266063.
  - This is a study discussing the role of PRRT in the management of small intestine neuroendocrine tumors.

## Data Availability

No datasets were generated or analysed during the current study.
